# Radiation-Induced Optical Coherence Tomography Angiography Retinal Alterations in Patients With Nasopharyngeal Carcinoma

**DOI:** 10.3389/fmed.2020.630880

**Published:** 2021-02-03

**Authors:** Zijing Li, Zongyi Zhan, Jianhui Xiao, Yuqing Lan

**Affiliations:** ^1^Department of Ophthalmology, Sun Yat-sen Memorial Hospital, Sun Yat-sen University, Guangzhou, China; ^2^Guangdong Provincial Key Laboratory of Malignant Tumor Epigenetics and Gene Regulation, Sun Yat-sen Memorial Hospital, Sun Yat-sen University, Guangzhou, China

**Keywords:** optical coherence tomography angiography, radiation encephalopathy, radiation retinopathy, radiation optic neuropathy, nasopharyngeal carcinoma

## Abstract

**Aim:** The aim of the study was to investigate the early neurovascular alterations of the retina in radiation encephalopathy (RE) patients with normal-ranged visual acuity after radiotherapy for nasopharyngeal carcinoma.

**Methods:** Fifty-five RE patients and 54 healthy age-matched subjects were enrolled in this retrospective cross-sectional case–control study. The best corrected visual acuity (LogMAR) of the included eye should not be more than 0. The vessel density and thickness of different locations in the retina were acquired automatically using optical coherence tomography angiography (OCTA). The data were then compared between the RE patients and the controls. The location included the whole retina, the superficial vascular plexus (SVP)/the ganglion cell complex (GCC), the deep vascular plexus (DVP), and the choroid in the macular area, as well as the inside disc and peripapillary area in the optic nerve head (ONH). The risk factors in OCTA retinal impairments were analyzed using a backward multiple linear regression. The relationships between mean deviation (MD) and pattern standard deviation (PSD) in the visual field (VF) and the OCTA parameters were also analyzed in RE patients.

**Results:** The vessel density of the GCC was significantly reduced in RE patients compared with controls (*p* = 0.018), and the reductions were mainly shown in the parafoveal (*p* = 0.049) and perifoveal fields (*p* = 0.006). The thickness of the GCC was correspondingly reduced (whole image GCC mean thickness: *p* = 0.044; parafoveal thickness: *p* = 0.038; perifoveal thickness: *p* = 0.038). In addition, the sub-foveal choroidal thickness (*p* = 0.039) was also reduced in RE patients. The vessel density of the GCC (*R*^2^ = 0.643) and DVP (*R*^2^ = 0.777) had a significant positive correlation with high-density lipoprotein cholesterol (HDL-C) and apolipoprotein A1 (ApoA1) and had a significant negative correlation with age (GCC: HDL-C, β = 29.89, *p* = 0.005; ApoA1, β = 78.92, *p* = 0.002; age, β = −0.886, *p* = 0.001; DVP: HDL-C, β = 40.09, *p* = 0.003; ApoA1, β = 62.65, *p* = 0.013; age, β = −1.31, *p* = 0.001). The vessel density of the GCC also had a significant negative correlation with apolipoprotein B (ApoB) (β = −32.18, *p* = 0.006). In the VF, MD had a significant positive correlation with the vessel density inside disc (*R*^2^ = 0.241, β = 0.304, *p* = 0.045), whereas PSD showed a significant negative correlation with the vessel density inside disc and the average GCC thickness, respectively (*R*^2^ = 0.437; vessel density inside disc, β = −0.358, *p* = 0.004; average GCC thickness, β = −0.510, *p* < 0.001).

**Conclusion:** With the aid of OCTA, we found that neurovascular alterations of the retina may exist in RE patients with normal-ranged visual acuity. Herein, we suggest the implementation of OCTA to assist ophthalmologists in the early detection and consistent monitoring of radiation-related eye diseases to avoid delayed diagnosis.

## Introduction

Nasopharyngeal carcinoma (NPC) has a higher incidence in Southeast Asia, particularly in South China (1). Currently, the most effective treatment against NPC is radiotherapy (RT) (2). However, radiation can affect the inferior temporal lobe of the brain, one of the adjacent tissues, causing radiation encephalopathy (RE) (3). RE is a delayed complication that is irreversible, severe, and progressive; it causes headaches, dizziness, and even dementia or mental disorders (1). Its 5-year incidence has been reported to be 16–34.9%; therefore, it becomes a principal factor affecting NPC patients' quality of life even when prolonged survival was attained (4, 5). Impaired visual function is a vital issue for RE patients; however, the early onset of this complication is asymptomatic, leading to late diagnosis. It is not until severe eye complications, such as radiation optic neuropathy (RON) and radiation retinopathy (RR), are presented that patients are properly diagnosed and receive treatment. Therefore, it is vital to assess RE patients' visual function when they are referred to the clinic even if they are asymptomatic, which may slow down the process of visual impairment and prevent visual loss.

Severe radiation-induced eye complications, such as RON and RR, are characterized by irreversible neural and microvascular impairments (6, 7). Most patients with RON or RR in previous studies were because of choroidal melanoma (CM), and not because of NPC. In these studies, the main tests performed on patients were optical coherence tomography (OCT) and fluorescein fundus angiography (FFA) (8–10). However, the visual function abnormalities, especially the microvascular alterations, were rarely studied in post-RT NPC patients without visual impairment. One reason could be the lower incidence of NPC in foreign countries. Other important reasons are that OCT cannot capture vessel network status and FFA is an invasive diagnostic technique. Intravenous dyes injection used in FFA may cause severe anaphylaxis; therefore, patients with serious systemic diseases may have lower tolerance toward the examination (11, 12). Recently, a cross-sectional real-time imaging machine, optical coherence tomography angiography (OCTA), a combination of traditional OCT and FFA, has been introduced to assist ophthalmologists in the detection of subtle microvascular changes in the retina and different kinds of retinal diseases, such as radiation-induced retinal diseases (13–16). OCTA not only can provide high-resolution images of each layer of the retina but also can quantify the retinal microvascular networks synchronously in a safe, non-invasive method, all without the use of exogenous dyes.

Herein, the major purpose of this study was to use OCTA to investigate the neurovascular alterations of the retina in RE patients with normal-ranged visual acuity after RT for NPC. Moreover, the relationships between visual field (VF) and OCTA neurovascular measurements were also analyzed in the RE group.

## Methods and Subjects

### Ethical Approval

All procedures followed were in accordance with the ethical standards of the responsible committee on human experimentation (institutional and national) and with the Helsinki Declaration of 1975, as revised in 2008 (5). Informed consent was obtained from all patients included in the study. Approval (approval number: 2017-06) for the study was obtained from the ethics committee of Sun Yat-sen Memorial Hospital, Sun Yat-sen University.

### Subjects

Fifty-five RE patients were recruited from the neurology and ophthalmology departments of Sun Yat-sen Memorial Hospital between January 2017 and January 2019 in this retrospective cross-sectional clinical study. Fifty-four healthy age-matched subjects were included as controls. The major inclusion criteria included a history of RT due to NPC and a diagnosis of RE in the patients. A diagnosis of RE was provided by a neurologist. The eye laterality of the affected cerebrum side was chosen, and if bilateral RE existed, the eye laterality of the more serious cerebrum side was selected. Thorough ophthalmic examinations, including best corrected visual acuity (BCVA), refractive error, intraocular pressure (IOP), axial length, dilated fundus examination, visual evoked potential (VEP), 30-2 VF testing (Carl Zeiss Meditec, Inc., Berlin, Germany), and OCTA (Optovue, Inc., CA, USA), were performed in RE patients when a diagnosis of RE was confirmed. IOP was measured using the Canon TX-20 non-contact tonometer (Canon Inc., Tokyo, Japan). VF testing was performed by two trained optometrists. The test was repeated when unreliable indices existed or patient had not understood the instructions during any step of the test procedure. Unreliable indices included a false positive/negative error score of more than 10% or a fixation loss score of more than 15% (17). In healthy controls, the above ophthalmic examinations were also performed with the exception of VEP and 30-2 VF testing. The BCVA (LogMAR) of the included eye should not be more than 0. All examinations were performed by experienced ophthalmologists. General characteristics including age, gender, years after RT, and complications after RT were recorded. The exclusion criteria were as follows: (1) diabetes mellitus, uncontrollable hypertension, and other serious systemic diseases; (2) glaucoma, uveitis, and other retinal diseases; (3) an axial length of the eye greater than 26, spherical equivalent outside the range between +3 and −3 diopters, or lens opacity that affected imaging; and (4) a history of intraocular surgery.

### RT Protocol

All RE patients had a history of external beam radiation therapy (EBRT). An EBRT setup, delivered in 2 Gy per day and 5 days per week, mainly consisted of opposing lateral photon fields (6–8 MV) to treat the nasopharynx and the upper neck. During the RT process, a cerrobend was applied to restrict the radiation area. The overall dose for all patients was about 65 Gy (60–75 Gy) to the nasopharyngeal primary site and 70 Gy to the metastatic lymph nodes.

### RE Lesion Volume Measurements

**Figure 1:**
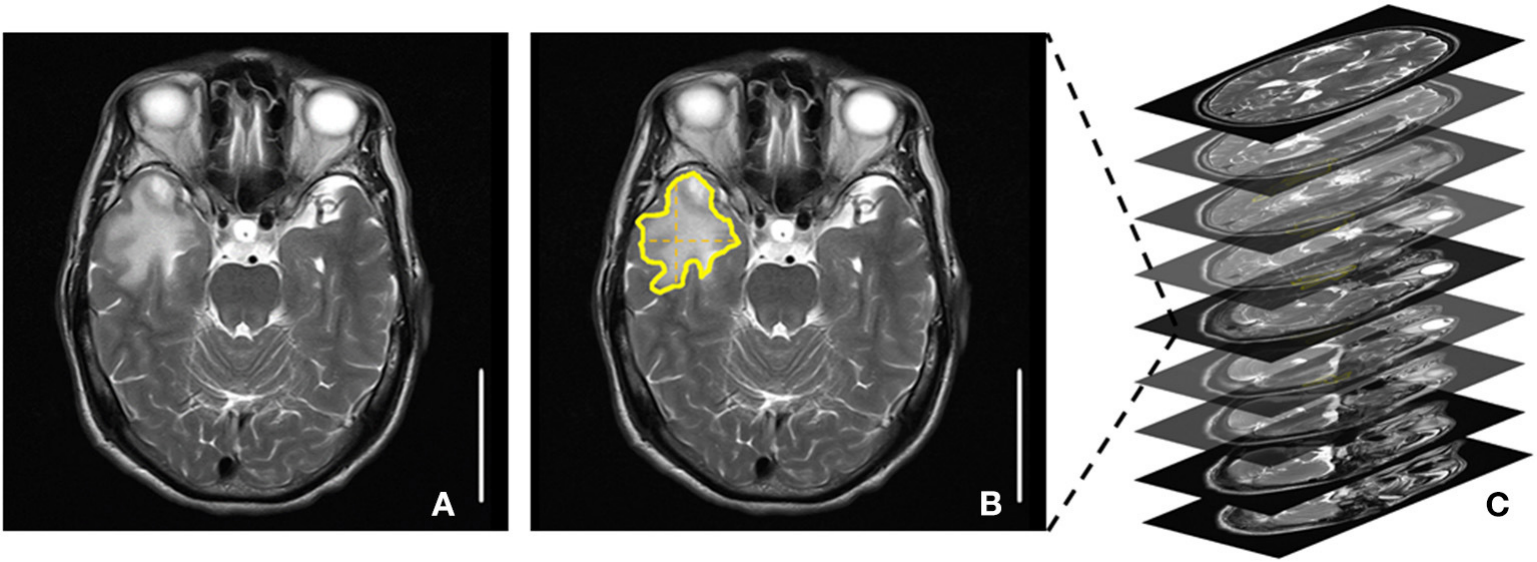
Radiation encephalopathy lesion volume measurements. **(A)** A T2 magnetic resonance imaging (MRI) image with the largest lesion area. **(B)** The largest lesion area: area surrounded by yellow lines. **(C)** The number of T2 MRI images with lesions. The RE lesion volume = the largest lesion area × the number of T2 MRI images with lesions.

The magnetic resonance imaging (MRI) images of RE patients were acquired. The RE lesion volume was detected by using T2-weighted fluid-attenuated inversion recovery and was independently assessed by a radiologist. The RE lesion volume = the largest lesion area × the number of T2 MRI images with lesions (Figure 1). A radiologist identified the outline of the largest lesion area (vertical and lateral diameter) manually and semiautomatically using Photoshop CS6 (Adobe, CA, USA). The number of T2 MRI images with lesions was roughly counted from all the T2 MRI images with lesions. A larger RE lesion volume represents a higher level of RE severity.

### OCTA Imaging and Statistical Analysis

**Figure 2:**
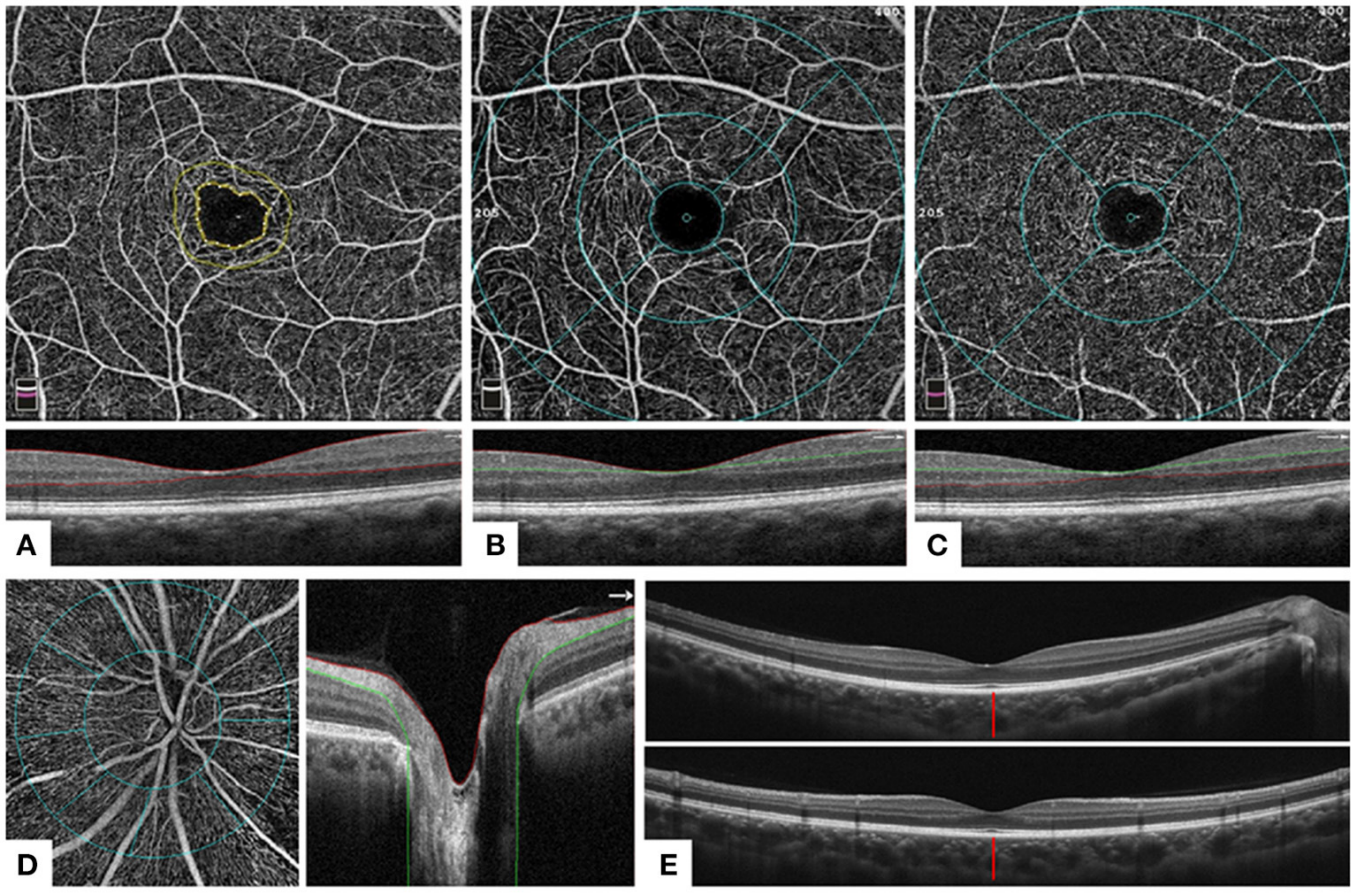
Optical coherence tomography and optical coherence tomography angiography measurements. **(A)** A 6 × 6 mm OCTA image (above) of the corresponding layer in the macular area of the whole inner retina [below: a horizontal OCT B-scan of internal limiting membrane (ILM)–outer plexiform layer (OPL), area between two red lines]. Foveal avascular zone (FAZ): area surrounded by the inner yellow line. **(B)** A 6 × 6 mm OCTA image (above) of the corresponding layer of the macular area in the superficial retina [below: horizontal OCT B-scan of ILM–inner plexiform layer (IPL), area between red and green lines]. Foveal area: area surrounded by the inner blue ring (ring diameter = 1 mm); parafoveal area: area between the middle (ring diameter = 3 mm) and inner blue rings; perifoveal area: area between the outer (ring diameter = 6 mm) and middle blue rings. The four quarters were divided by four blue lines. **(C)** A 6 × 6 mm OCTA image (above) of the corresponding layer of the macular area in the deep inner retina [below: horizontal OCT B-scan of IPL–OPL, area between green and red lines]. Sections division was the same as **(B)**. **(D)** A 4.5 × 4.5 mm OCTA image (left) of the corresponding layer in the radial peripapillary capillaries layer (right: horizontal OCT B-scan of ILM–retinal nerve fiber layer, area between red and green lines) of the optic nerve head (ONH). Peripapillary: area surrounded by the inner blue ring; inside disc: area between two blue circles (the width is 1 mm). Eight sectors were divided by eight blue lines. **(E)** A horizontal (above) and a vertical (below) OCT B-scan of the retina. The distance of the red line: sub-foveal choroidal thickness, defined as the distance between the outermost edge of the retinal pigment epithelium and the sclera–choroidal border.

OCTA images were obtained using AngioVue software 2.0 of the RTVue XR Avanti device (Optovue, Inc., CA, USA). A speed of 70,000 A scans per second and a split-spectrum amplitude-decorrelation angiography (SSADA) algorithm were applied in the scan. Images were excluded when the scan quality was < 6 or obvious artifacts were detected by a senior ophthalmologist's thorough check. The vessel density of the macular area and the optic nerve head (ONH) area was assessed in Angio Retina mode (6 × 6 mm) and Angio Disc mode (4.5 × 4.5 mm), respectively. Foveal avascular zone (FAZ) was automatically obtained based on the superficial vascular plexus (SVP) image in Angio Retina mode (6 × 6 mm). The retinal thickness and retinal nerve fiber layer (RNFL) thickness were assessed using the Retina Map mode and ONH mode. The average ganglion cell complex (GCC) thickness, global loss volume (GLV), and focal loss volume (FLV) were calculated in the GCC mode. The above measurements were automatically exported from AngioVue 2.0. In addition, sub-foveal choroidal thickness (SFCT) defined as the distance between the outermost edge of the retinal pigment epithelium (RPE) and the sclera–choroidal border was measured manually. The average SFCT was defined as the average value of the horizontal and vertical SFCT. Major OCTA measurements were explained in Figure 2.

The statistical analyses were performed using SPSS 24.0 (SPSS Inc., IL, USA). Independent two-tailed Student's *t*-tests were performed to compare normally distributed data between RE patients and the controls. Categorical variables were analyzed with a chi-squared test. In RE patients, backward multiple linear regression analyses were performed between the major OCTA measurements [whole image vessel density/thickness of the GCC/deep vascular plexus (DVP) and the radial peripapillary capillary plexus] and the other factors including age, the RE lesion volume, radiation dose, RE symptoms onset time, systolic and diastolic blood pressure, HbA1c, total cholesterol (TC), triglyceride (TG), high-density lipoprotein cholesterol (HDL-C), low-density lipoprotein cholesterol (LDL-C), apolipoprotein A1 (ApoA1), and apolipoprotein B (ApoB) in order to assess the risk factors of retinal impairments. Moreover, a backward multiple linear regression was applied between the above OCTA measurements and the VF parameters including mean deviation (MD) and pattern standard deviation (PSD).

## Results

### Patients' Characteristics

Fifty-five RE patients and 54 age-matched healthy controls were included in this study. There was no statistically significant difference with regard to age, gender, hypertension, diabetes mellitus, eye laterality, or BCVA between RE patients and the controls (Table 1). The IOP in both groups was in the normal range. In the VEP test, 23.64% (13/55) of RE patients had a prolonged latency period, and 21.82% (12/55) had a reduced amplitude. Other common complications after RT included strabismus, headache, dizziness, dysarthria, dysphagia, decreased hearing, tinnitus, and limb numbness. Detailed statistical data are shown in Table 1.

**Table 1:** Patient characteristics.

	**RE**	**Control**	***p***
Patients (n)	55	54	NA
Mean age, range (years)	47.78 ± 8.94	46.78 ± 9.33	0.57
Male: female	43:12	42:12	0.96
Hypertension (yes/no)	14/41	19/35	0.269
Systolic blood pressure (mmHg)	124.74 ± 24.35	NA	NA
Diastolic blood pressure (mmHg)	81.41 ± 16.60	NA	NA
Diabetes mellitus (yes/no)	2/52	1/52	1.000
HbA1c (%)	5.59 ± 0.40	NA	NA
Serum lipid			
TC (mmol/L)	4.93 ± 1.42	NA	NA
TG (mmol/L)	1.25 ± 0.94	NA	NA
HDL-C (mmol/L)	1.13 ± 0.27	NA	NA
LDL-C (mmol/L)	3.15 ± 1.11	NA	NA
ApoA1 (g/L)	1.13 ± 0.18	NA	NA
ApoB (g/L)	0.92 ± 0.29	NA	NA
Radiation dose of the nasopharynx (Gy)	64.75 ± 2.35	NA	NA
RE symptoms onset time (years after radiotherapy)	6.88 ± 4.65	NA	NA
RE lesion volume	108.55 ± 208.37	NA	NA
Laterality (right: left)	26:29	27:27	0.78
LogMAR BCVA	−0.06 ± 0.07	−0.06 ± 0.08	0.748
IOP (mmHg)	15.10 ± 2.68	15.50 ± 2.72	0.44
Other complications			
Strabismus (%)	1.82% (1/55)	NA	NA
Headache and dizziness (%)	10.91% (6/55)	NA	NA
Dysarthria (%)	9.09% (5/55)	NA	NA
Dysphagia (%)	12.73% (7/55)	NA	NA
Decreased hearing and tinnitus (%)	14.55% (8/55)	NA	NA
Limb numbness (%)	3.64% (2/55)	NA	NA

RE, radiation encephalopathy; NA, not available; TC, total cholesterol; TG, triglyceride; HDL-C, high-density lipoprotein cholesterol; LDL-C, low-density lipoprotein cholesterol; ApoA1, apolipoprotein A1; ApoB, apolipoprotein B; BCVA, best corrected visual acuity; IOP, intraocular pressure. *p < 0.05.

### OCTA Findings

With regard to vessel density, the whole image density of the macular area in the GCC [internal limiting membrane–inner plexiform layer (ILM–IPL)] was significantly reduced in RE patients compared with the control group (48.55 ± 3.22 vs. 49.94 ± 2.63%, *p* = 0.018), whereas the density in the deep layer [inner plexiform layer–outer plexiform layer (IPL–OPL)] and the density near the ONH were not significantly reduced in RE. Moreover, no significant difference was shown in the FAZ between two groups, but the average FAZ area in the RE group was larger than that in the control group. Detailed data are shown in Table 2.

**Table 2:** Major vessel measurements in OCTA between RE and controls.

	**RE**	**Control**	***p***
**SVP in the macular area: ILM–IPL**			
Whole image VD (%)	48.55 ± 3.22	49.94 ± 2.63	^*^0.018
Superior VD (%)	48.82 ± 3.32	50.23 ± 2.74	^*^0.021
Inferior VD (%)	48.27 ± 3.35	49.67 ± 2.69	^*^0.022
FAZ (mm^2^)	0.32 ± 0.10	0.29 ± 0.10	0.098
Foveal VD (ring diameter = 1 mm) (%)	17.46 ± 6.00	18.80 ± 6.87	0.296
Parafoveal VD (ring diameter = 3 mm) (%)	50.85 ± 3.96	52.23 ± 3.01	^*^0.049
Perifoveal VD (ring diameter = 6 mm) (%)	48.87 ± 3.50	50.60 ± 2.73	^*^0.006
**DVP in the macular area: IPL–OPL**			
Whole image VD (%)	49.70 ± 5.98	50.82 ± 4.71	0.296
**RPCP in the ONH**			
Whole image VD (%)	49.33 ± 3.21	50.03 ± 2.80	0.244
Inside disc VD (%)	50.60 ± 5.60	52.32 ± 4.72	0.090
peripapillary VD (%)	51.24 ± 3.90	52.18 ± 3.25	0.181

*RE, radiation encephalopathy; SVP, superficial vascular plexus; ILM, internal limiting membrane; IPL, inner plexiform layer; VD, vessel density; FAZ, foveal avascular zone; DVP, deep vascular plexus; OPL, outer plexiform layer; RPCP, radial peripapillary capillary plexus; ONH, optic nerve head*.

* *p < 0.05*.

Significantly reduced thickness of the GCC (ILM–IPL) and outermost retinal layers [retinal pigment epithelium–Bruch's membrane (RPE–BRM)] was found in RE patients (GCC: 97.86 ± 9.85 vs. 101.05 ± 5.88 μm, *p* = 0.044; RPE–BRM thickness: 9.87 ± 2.21 vs. 11.22 ± 4.12 μm, *p* = 0.049). SFCT and inferior GCC thickness were also decreased in RE patients (SFCT: 262.22 ± 64.56 vs. 290.48 ± 69.52 μm, *p* = 0.039; inferior GCC thickness: 94.23 ± 10.35 vs. 97.70 ± 5.20 μm, *p* = 0.033). Besides, GLV was also noticeably increased in RE patients (4.31 ± 5.36 vs. 1.96 ± 1.84%, *p* = 0.003). Detailed data are shown in Table 3.

**Table 3:** Major thickness measurements in OCTA between the RE and controls.

	**RE**	**Control**	***p***
**SVP + DVP in the macular area: ILM–RPE (μm)**			
Whole image mean thickness (μm)	293.86 ± 17.12	296.08 ± 10.74	0.425
Foveal thickness (ring diameter = 1 mm) (μm)	241.93 ± 23.70	244.95 ± 19.75	0.476
**SVP in the macular area: ILM–IPL**			
Whole image GCC mean thickness (μm)	97.86 ± 9.85	101.05 ± 5.88	^*^0.044
Superior GCC thickness (μm)	95.74 ± 8.05	97.76 ± 5.71	0.143
Inferior GCC thickness (μm)	94.23 ± 10.35	97.70 ± 5.20	^*^0.033
FLV (%)	1.52 ± 2.64	0.81 ± 0.82	0.065
GLV (%)	4.31 ± 5.36	1.96 ± 1.84	^*^0.003
Foveal thickness (ring diameter = 1 mm) (μm)	48.87 ± 9.08	51.71 ± 8.60	0.101
Parafoveal thickness (ring diameter = 3 mm) (μm)	103.29 ± 13.45	107.65 ± 6.81	^*^0.038
Perifoveal thickness (ring diameter = 6 mm) (μm)	97.32 ± 10.65	100.93 ± 6.54	^*^0.038
**DVP in the macular area: IPL–OPL**			
Whole image mean thickness (μm)	48.87 ± 9.08	48.87 ± 9.08	0.185
**RPE–BRM mean thickness (μm)**	9.87 ± 2.21	11.22 ± 4.12	^*^0.049
**Peripapillary RNFL (μm)**	113.74 ± 17.31	117.40 ± 17.82	0.290
**Sub-foveal choroidal thickness (μm)**	262.22 ± 64.56	290.48 ± 69.52	^*^0.039

*RE, radiation encephalopathy; SVP, superficial vascular plexus; DVP, deep vascular plexus; ILM, internal limiting membrane; RPE, retinal pigment epithelium; IPL, inner plexiform layer; GCC: ganglion cell complex, includes retinal nerve fiber layer, ganglion cell layer, and inner plexiform layer; FLV, focal loss volume, the sum of fractional deviation in the region where there is significant focal loss (pattern deviation more than 1.65 standard deviations below the normal average); GLV, global loss volume, the sum of fractional deviation in areas where fractional deviation is negative; OPL, outer plexiform layer; BRM, Bruch's membrane; RNFL, retinal nerve fiber layer. RNFL thickness, GCC thickness, FLV, and GLV were measurements in retinal ganglion cells assessment*.

* *p < 0.05*.

### Risk Factors of Retinal Impairments

The backward multiple linear regression analyses between the major OCTA measurements and the other factors (age, the RE lesion volume, radiation dose, RE symptoms onset time, systolic and diastolic blood pressure, HbA1c, TC, TG, HDL-C, LDL-C, ApoA1, and ApoB) revealed that the vessel density of the GCC and DVP was correlated to these factors. The vessel density of the GCC had a significant positive correlation with TG, HDL-C, and ApoA1 and had a significant negative correlation with age, LDL-C, and ApoB (*R*^2^ = 0.643; TG, β = 2.06, *p* = 0.014; HDL-C, β = 29.89, *p* = 0.005; ApoA1, β = 78.92, *p* = 0.002; age, β = −0.886, *p* = 0.001; LDL-C, β = −23.91, *p* = 0.003; ApoB, β = −32.18, *p* = 0.006). Similarly, the vessel density of the DVP showed a significant positive correlation with HDL-C, ApoA1, diastolic blood pressure, and TG and showed a significant negative correlation with age and TC (*R*^2^ = 0.777; HDL-C, β = 40.09, *p* = 0.003; ApoA1, β = 62.65, *p* = 0.013; diastolic blood pressure, β = 0.139, *p* < 0.022; TG, β = 7.17, *p* = 0.003; age, β = −1.31, *p* = 0.001; TC, β = −20.76, *p* = 0.007). However, the retinal thickness of the macular, peripapillary vessel density, and RNFL thickness did not show any correlation with the other factors. Thus, it seemed that TG, HDL-C, ApoA1, and diastolic blood pressure were major protective factors of retinal vessels, whereas age, LDL-C, ApoB, and TC were risk factors of retinal impairments.

### Relationship Between VF and OCTA

**Figure 3:**
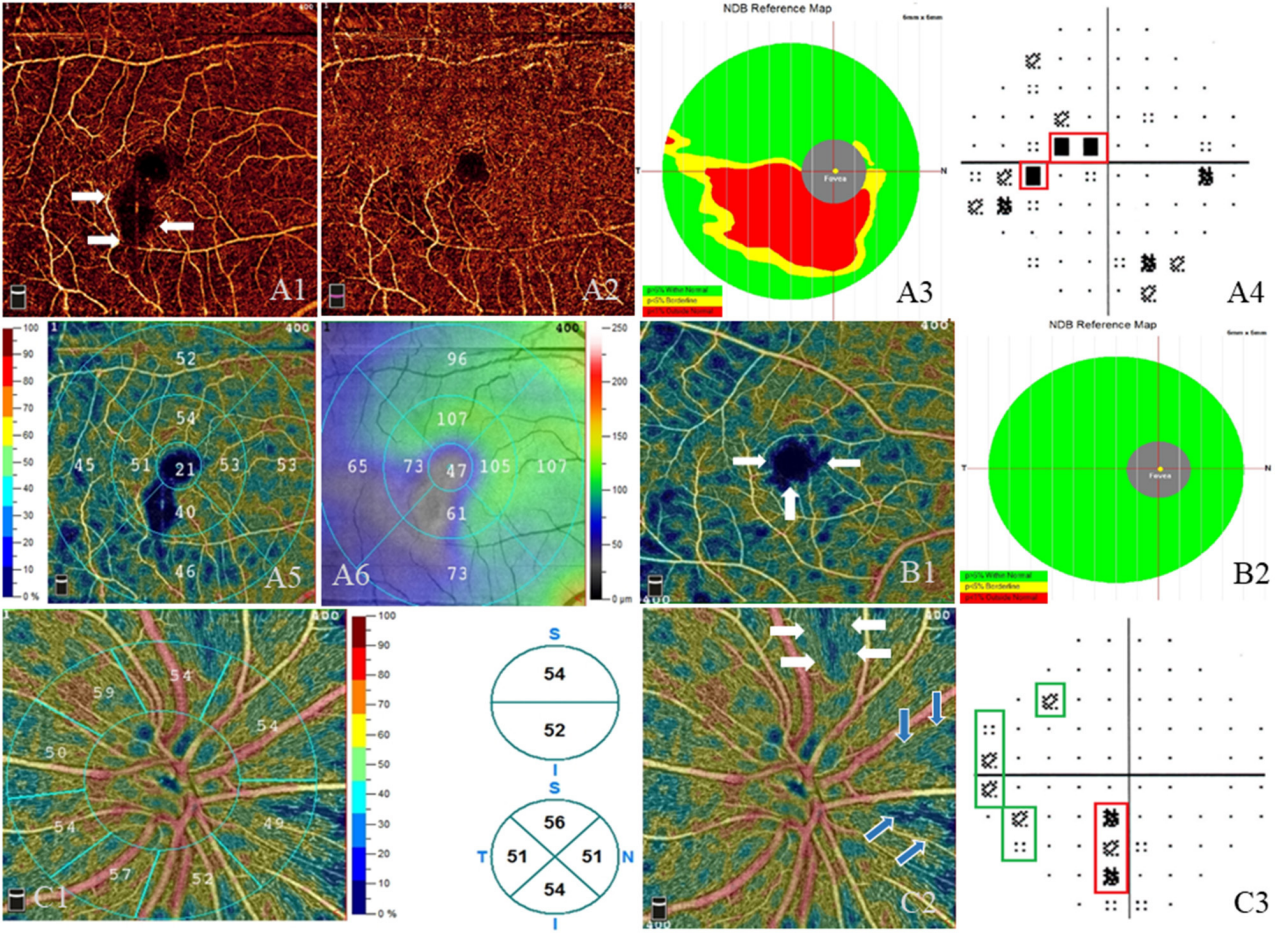
Visual field defects and impairment in the macular and optic nerve head (ONH) areas. **(A–C)** Three different radiation encephalopathy patients. **(A)** The right eye of a radiation encephalopathy patient (RE-1). **(A1)** Lower vessel density (white arrows) adjacent to the macula in the superficial vascular plexus (SVP, from internal limiting membrane to inner plexiform layer). **(A2)** Less obvious lower vessel density in the corresponding location in the deep vascular plexus (from inner plexiform layer to outer plexiform layer). **(A3)** Significantly reduced ganglion cell complex thickness (red area) in the corresponding location. **(A4)** The corresponding central scotoma (red line) in the pattern standard deviation (PSD) map. **(A5)** The vessel density of different sectors in the SVP layer and corresponding lower vessel density in the inferior. **(A6)** Retinal thickness of different sectors in the SVP layer and corresponding thinner thickness in the inferior and the temporal. **(B)** The right eye of RE-2. **(B1)** Enlarged foveal avascular zone (white arrows) and diffused vessel density reduction (dark blue area) in the SVP layer. **(B2)** Normal ganglion cell complex thickness in the corresponding location. **(C)** The right eye of RE-3. **(C1)** Left: the vessel density of different sectors in the radial peripapillary capillary plexus (RPCP) layer, lower vessel density in the nasal inferior; right: the vessel density of hemifield (up) and of quadrant (down) in the RPCP layer. **(C2)** Lower RPCP (white arrows and blue arrows) near the ONH. **(C3)** The corresponding visual field defect (red line and green lines) in the PSD map.

In the RE group, the average MD and PSD was −2.22 ± 3.14 and 2.43 ± 2.08 dB, respectively. An abnormal VF was defined as not less than three adjoining test spots that had a sensitivity of < 2% of the normal average in the PSD map. Of the patients, 40% (22/55) had a normal VF, whereas 60% (33/55) had an abnormal VF. A backward multiple linear regression was applied between the above OCTA measurements and the VF parameters (MD and PSD) in RE patients. MD had a significant positive correlation with the vessel density inside disc (*R*^2^ = 0.241, β = 0.304, *p* = 0.045), whereas PSD showed a significant negative correlation with the vessel density inside disc and the average GCC thickness, respectively (*R*^2^ = 0.437; vessel density inside disc, β = −0.358, *p* = 0.004; average GCC thickness, β = −0.510, *p* < 0.001). No significant correlation was observed between PSD (or MD) and the rest of the measurements (i.e., FAZ, macular vessel density, macular thickness, peripapillary RNFL, SFCT). The VF defects were correspondent to the location where the vessel density and GCC thickness were reduced (Figure 3).

## Discussion

To date, few studies have been done on OCTA imaging of RE patients, especially in NPC patients who had a history of RT treatments. Therefore, the purpose of this study is to investigate the neurovascular differences of the retina between RE patients with normal-ranged visual acuity and the control group. In our study, we revealed that the retinal structure was affected in RE patients even before obvious visual function impairments emerged, as indicated by the reduced vessel density and thickness of the retina.

According to previous studies, significant reduction of the vessel density of the macular area and ONH was shown in patients after RT for CM (13, 18, 19). Our findings were similar to these studies; however, some differences were noted. In previous studies, the vessel densities of the SVP and DVP were significantly reduced, but, in our study, the vessel density in the RE group was observed to be significantly reduced mainly in the SVP, whereas it was only minimally reduced in the DVP.

There are some explanations for the above phenomenon. One explanation is that the inapparent reduction in the DVP was partly related to the disease progression. Radiation impaired the vascular endothelial cell (VEC) of venules, arterioles, and capillaries, ultimately blocking the vessels and decreasing the densities in both the SVP and DVP (20). The SVP has a large number of venules and arterioles and only has a small number of capillaries, whereas the DVP has more abundant capillary networks. Before the vessels were blocked, the aggregation of blood cells dilated the vessel diameter in the capillary networks and increased the perfusion density (21). Since the average density may depend on the ratio of blocked vessels to dilated vessels, if the number of dilated vessels was relatively larger, it may counteract the reduced vessel density of the DVP to some degree.

Another possible explanation is layer segmentation errors caused by irregular boundaries and shapes of the capillary networks. Projection artifacts from other layers may also be a reason for the inapparent reduction in the DVP. To avoid projection artifacts, Sellam et al. (22) considered using a full vascular network instead of splitting them into two plexuses when they studied OCTA imaging of radiation maculopathy.

Moreover, the tumor type, as well as the RT methods, may affect the vessel density (14, 23, 24). As CM is a disease of the choroid, factors, such as proinflammatory cytokines and vascular endothelial growth factor (VEGF), released by tumors could accelerate the development of retinal ischemic changes (14, 23). Besides, plaque radiotherapy (PR) was applied in CM patients, whereas EBRT was performed in NPC patients. In PR, the radioactive sources were closer to the eyes and consequently resulted in a more direct radiation effect than that of EBRT, which also explained the possible reason for obvious ischemia observed in post-RT CM patients (24).

Another finding in the RE cohort was that the GCC vessel density reductions were mainly shown in the parafoveal and perifoveal fields. As the fovea lacks the structure of the GCC (25) and vessels and the radiation initially damages the SVP according to our study, the radiation effect on the vessel density of the foveal area may be less than that of the other fields. Besides, due to low perfusion densities, a significantly and correspondingly reduced thickness of the parafoveal and perifoveal fields was shown in RE patients. The corresponding relationship between vessel density and thickness in RE-related retinal changes was similar to that in glaucoma. Takusagawa et al. (26) revealed that reduced vessel density, as well as corresponding GCC thickness, was noticed even in perimetric glaucoma. Recently, Parrozzani and Veverka proposed a clinical grading of RON and RR using OCTA (27, 28). According to their grading, some asymptomatic RE patients can be considered as mild or even moderate RON and RR. However, impairments in these grades may not cause decreased visual acuity; therefore, mild and even moderate RON or RR may be asymptomatic at the early onset of RE and might be easily overlooked. These evidences suggest that OCTA may be a promising tool in asymptomatic RE-related retinal changes screening.

The significant increment of GLV suggested a primarily diffused impairment in the retinal ganglion cells. Besides, interestingly, reduced GCC thickness, which means neural impairment, was prominent in the inferior section in the RE group. This might be due to the close proximity of the radiation area, as the nasopharynx is located below the horizontal surface of the eyeball (29).

The most interesting finding in the risk factor analyses was that serum lipid-related items were much related to retinal vessel impairment. A higher level of ApoB and a lower level of ApoA1 may contribute to the risk of RE-related retinal diseases. ApoB releases proinflammatory factors and is highly involved in atherogenesis, whereas ApoA1 has anti-inflammatory effect and initiates reverse cholesterol transport from the vessels to the liver (30). A high ApoB/ApoA1 ratio has been considered as risk factors in ischemic stroke, carotid stenosis, and other vascular diseases, which was consistent with our present study in some degrees (31, 32). However, factors about RE severity and radiation dose were not related to the OCTA parameters, which suggested that a number of factors, such as treatment duration, interval between RT and OCTA, and distance between RE lesions and optic nerve, should be consider in future prospective research.

Unlike glaucoma and optic neuritis, most of the VF defects patterns were irregular and lacked distinguishable characteristics. Ozkaya et al. (33) found that VF and contrast sensitivity were significantly affected with a mean dose of more than 50 Gy in NPC patients. According to Ferguson (34), no significant correlation for other risk factors was identified in RON when a lower radiation dosage was applied in EBRT. The mean dose of our patients reached ~65 Gy, much higher than the threshold presented by Ozkaya. That might account for the fact that 60% of RE patients had abnormal VF. Consequently, the dose of radiation may be a vital factor in causing varied VF defects patterns. The radiation area, location, and tumor size should be taken into consideration in further research in regard to radiation dose and their side effects on VF.

In our study, although vessel density inside disc was associated with both MD and PSD, stronger correlation was seen in the latter. Besides, the goodness-of-fit between OCTA measurements and PSD (*R*^2^ = 0.437) was better than that between OCTA measurements and MD (*R*^2^ = 0.241). Similarly, Liu and his colleagues (35) also found that peripapillary vessel density was more strongly correlated with PSD than with MD in glaucomatous patients. The effect of dioptric media opacities and small pupils was excluded in PSD, and PSD may be more sensitive and accurate in detecting focal VF damages. However, in Yarmohammadi and Hwang's studies (36, 37), OCTA measurements were more highly correlated with MD, indicating that the varied assessment methods of vascular network, VF defects pattern, and severity, as well as other systemic diseases, could affect retinal vasculature measurements, possibly leading to inconsistency in each study.

Consistent with previous research about glaucoma (26, 35), we noticed that besides vessel density near the ONH, GCC thickness was also significantly associated with the severity of VF damage. The VF damage locations corresponded to the reduction of vessel density and GCC thickness. Using OCTA, the severity and location of the VF defects may be roughly speculated. Therefore, OCTA may be suitable for monitoring the neurovascular alterations in both glaucoma and radiation eye diseases.

The major limitation of our study was that it was a retrospective study. Some data were not complete enough. In addition, many confounding factors may affect the results. For example, retinal changes and complications secondary to chemotherapy may occur in different cancers, but these retinal complications were rare, and the dose in NPC was relatively lower than that in other cancers (38), thus may diminish the effect in some degree. Nevertheless, chemotherapy effects on retinal OCTA changes deserve further exploration. Another limitation was that it had a small sample size and large-scale longitudinal studies are waiting to be performed. Moreover, the detection area of OCTA was narrow, and the peripheral vascular changes could be easily neglected. A proper velocity of the blood cell, which was neither too fast nor too slow, was required in the detection, which may significantly affect vessel measurements.

## Conclusion

With the aid of OCTA, we found that neurovascular alterations of the retina may exist in RE patients with normal-ranged visual acuity. Herein, we suggest the implementation of OCTA to assist ophthalmologists in the early detection and consistent monitoring of radiation-related eye diseases to avoid delayed diagnosis.

## Data Availability Statement

The original contributions presented in the study are included in the article/supplementary material, further inquiries can be directed to the corresponding author/s.

## Ethics Statement

The studies involving human participants were reviewed and approved by the ethics committee of Sun Yat-sen Memorial Hospital, Sun Yat-sen University. The patients/participants provided their written informed consent to participate in this study. Written informed consent was obtained from the individual(s) for the publication of any potentially identifiable images or data included in this article.

## Author Contributions

ZL: data analysis and manuscript drafting. ZZ: data collection and image editing. JX and YL: manuscript design and manuscript polishment. All authors read and approved the final version of the manuscript.

## Conflict of Interest

The authors declare that the research was conducted in the absence of any commercial or financial relationships that could be construed as a potential conflict of interest.
